# Automatic generation of operation notes in endoscopic pituitary surgery videos using workflow recognition

**DOI:** 10.1016/j.ibmed.2023.100107

**Published:** 2023

**Authors:** Adrito Das, Danyal Z. Khan, John G. Hanrahan, Hani J. Marcus, Danail Stoyanov

**Affiliations:** a Wellcome/EPSRC Centre for Interventional and Surgical Sciences, University College London, United Kingdom; b National Hospital for Neurology and Neurosurgery, University College London, United Kingdom

**Keywords:** Computer vision, Image recognition, Operation report, Step recognition, Surgical AI, Workflow analysis

## Abstract

Operation notes are a crucial component of patient care. However, writing them manually is prone to human error, particularly in high pressured clinical environments. Automatic generation of operation notes from video recordings can alleviate some of the administrative burdens, improve accuracy, and provide additional information. To achieve this for endoscopic pituitary surgery, 27-steps were identified via expert consensus. Then, for the 97-videos recorded for this study, a timestamp of each step was annotated by an expert surgeon. To automatically determine whether a step is present in a video, a three-stage architecture was created. Firstly, for each step, a convolution neural network was used for binary image classification on each frame of a video. Secondly, for each step, the binary frame classifications were passed to a discriminator for binary video classification. Thirdly, for each video, the binary video classifications were passed to an accumulator for multi-label step classification. The architecture was trained on 77-videos, and tested on 20-videos, where a 0.80 weighted-*F*_1_ score was achieved. The classifications were inputted into a clinically based predefined template, and further enriched with additional video analytics. This work therefore demonstrates automatic generation of operative notes from surgical videos is feasible, and can assist surgeons during documentation.

## Highlights

• The first automatic generation of operation notes in endoscopic pituitary surgery.
• The first operation notes enriched with additional video analytics.
• A multi-label steps classifier for surgical steps recognition within a given video.

## 1 Introduction

Operation notes are the written documentation of an operation, providing details from basic patient identification to the procedural steps, and are important for patient care; clinical continuity; audit; research; education; and medico-legal processes [1]. However, they are often incomplete; inaccurate; lack detail; illegible; or late [1,2]. This is largely caused by human error, due to the ever-increasing administrative pressure on clinicians during surgery, and the limited time and resources available to them [1]. For example, a 2021 study of a hospital in Dublin found 80% of operation notes were incomplete, taking 10-minutes to write an operation note on mean-average [2].

In recent years, recording footage of a surgery has become more common [3], and utilising these recordings has been shown to add important information beyond what is documented in operation notes [4]. In particular, it was found that important surgical steps were missing in the operation notes compared to the surgical videos of laparoscopic cholecystectomy, such as the critical view of safety not reported in 25% of surgeries where it did occur in the video [4]. Thus, surgical video analysis is able to improve operation note accuracy and granularity [5]. This is unfortunately a labour and time intensive task, and so not feasible in the contemporary clinical environment [6,7]. However, automation has the potential to overcome these barriers [[6], [7], [8]], and assist surgeons when writing operation notes [5,6].

A surgical video can be broken down into several surgical phases, which in turn can be broken down into several more granular surgical steps [9,10]. In recent years, artificial neural networks (ANNs) have been shown to be an effective method in automating the recognition of these phases/steps within a video, with a focus on laparoscopic cholecystectomy due to the availability of publicly available annotated data [8,10]. It has also been achieved in videos of other surgeries, including endoscopic pituitary surgery [11,12]. The ANNs are able to predict the transition of one surgical phase/step to the next by using using a convolution neural network (CNN) for spatial recognition to classify which surgical phase/step a static frame of a recorded video belongs to [10,11]. Spatial-temporal recognition, such as using ANNs that utilise the temporal information in a video, or using statistical techniques that utilise phase/step and frame ordering are able to improve performance further [10,11].

In these papers, it is often discussed that the information provided by phase/step recognition can be utilised to automatically generate operation notes [6,8,10,11]. One such example is for laparoscopic cholecystectomy videos, where it was shown that an increased uncertainty of the ANN phase transition prediction was correlated with an adverse event, and hence this could be used as a flag during operation notes generation [13]. However, the surgical steps found in operation notes of endoscopic pituitary surgery are more granular, with 27-steps compared to the fewer 8-phases present in these studies [13,14]. Moreover, some steps occur concurrently with one another, changing the recognition task from a single-label classification problem to a multi-label classification problem [14].

To overcome this particular challenge, a three-stage architecture has been created. In the first stage, for each step, a CNN is used as a binary image classifier for each frame in the video (either the step is present in a frame or not). In the second stage, for each step, a “discriminator” is used as a binary video classifier (either the step is present in the video or not). This is done by implementing a numerical threshold on the frame classifications from the first stage: several discriminators were trailed. In the third stage, for each video, an “accumulator” is used as a multi-label step classifier. This is done by combining the binary video classifications from the second stage and ensuring predefined clinically-based relationships between the steps hold, such as a given step not being possible without another step also being present.

The final classifications are then fed into a predetermined clinically-based operation notes template, and further enriched with a 3-phases duration chart. This paper's contribution is therefore two-fold:

1. The first automatic generation of operation notes in endoscopic pituitary surgery using workflow recognition.
2. A novel three-stage architecture used as a multi-label steps classifier to determine which surgical steps are present within a given video.

## 2 Methods

### 2.1 Dataset

The 97-videos dataset of endoscopic pituitary surgery was collected from the National Hospital for Neurology and Neurosurgery (Queens Square, London, United Kingdom) between the 30th of August 2018 and the 20th of February 2021. This study was registered with the local governance committee, and all patients have provided written informed consent. Recordings were excluded if (i) the operation was a revision surgery within six months of the primary surgery or (ii) large sections were missing. The 97-videos have a median length of 74 minutes, with 15 having minor footage losses. The surgeries were recorded using a high-definition endoscope (Hopkins Telescope), with resolutions varying from 720p-2160p, at 25 frames per second (fps), and stored as mp4 files. For consistency and reduced computational time, the video resolutions were dropped to 720p, and converted to jpeg images at 1fps.

27 surgical steps were identified as key indicators for generating the operation notes. These steps were decided via a Delphi consensus of expert endoscopic pituitary surgeons, defined based on anatomical landmarks; surgical actions; and instrument usage [14]. Step to operation notes statement mappings are given in Table 1. For simplicity of terminology, when referring to a particular step, “S” for “surgical step” is added as a prefix to the step number. For this study “instruments” were identified as: S08 (drill); S11 (stealth pointer); S12 (doppler); S18 (surgiflo); S19 (biploar); and S20 (spongostan placement). These instruments are used to perform a “core” step, and therefore can occur simultaneously with this core step, although they are still considered as a separate surgical step for classification purposes.

**Table 1:** Surgical step to operation notes statement mapping. Phases and are defined in Ref. [14]. A step referred in a statement within square brackets [] signifies a continuation of the original statement with the bracketed step statement. A reliant step is one that requires a separate step, as given in the “reliant” column, to be present. Note S09 and S10 are two different steps that lead to the same operation notes statement (written across both lines).

Phase	Step	Category	Reliant	Operation notes statement
01	01	core	–	The middle and superior turbinates were laterally displaced using a freer elevator.
	02	core	–	The sphenoid ostium was identified [03].
	03	core	02	and opened using Kerrison's rongeurs.
	04	optional	–	The septum was then displaced [05] until the opposite ostium was seen.
	05	optional	–	and a partial posterior septectomy performed.
	06	core	–	The sphenoid sinus was opened, with removal of sphenoid septations [07] to expose the face of the sella. [08]
	07	core	–	and mucosa.
	08	instrument	–	A high-speed drill was required to achieve this.
02	09	core	–	The sella, carotid prominence, optic prominence, and optic-carotid recesses..
	10	core	–	..were then identified on both sides [11] [12].
	11	instrument	09/10	and confirmed using neuronavigation.
	12	instrument	09/10	and confirmed using a Micro Doppler probe.
	13	core	–	The sella was carefully opened using a rongeurs.
	14	core	–	A cruciate durotomy was performed using a retractable scalpel.
	15	core	–	The tumour was seen immediately on entering the sella and.
				.removed in a piecemeal fashion using currettes and pituitary rongeurs.
	16	core	–	The cleared pituitary fossa was visualised, and the diaphragm had descended.
03	17	core	18/19/20	Haemostasis was achieved with [18][19][20].
	18	instrument	17	a surgiflo.
	19	instrument	17	a bipolar cautery.
	20	instrument	–	and a spongostan placement.
	21	optional	–	A fat graft was harvested from the left lower quadrant of the abdomen and placed over the defect.
	22	optional	–	A MedPor implant was then sized and placed.
	23	optional	–	A fascia lata graft was then harvested, and placed over the construct.
	24	optional	–	Evicel was used.
	25	optional	–	Adherus dural sealant was applied.
	26	optional	–	Bismuth soaked ribbon gauze was then used to pack the nasal cavity and support the repair.
	27	core	–	Debris was cleared from the nasal cavity and choana.

All 97-videos were viewed by two expert surgeons and, by consensus, each surgical step was annotated with a timestamp. Within a video, a step may appear several times in non-consecutive frames, and these were also annotated with a timestamp. Moreover, the 27-steps vary in length (median range 1–11 minutes) and are not necessarily sequential, and so these variabilities will be needed to be accounted for during classification.

**Fig. 1:**
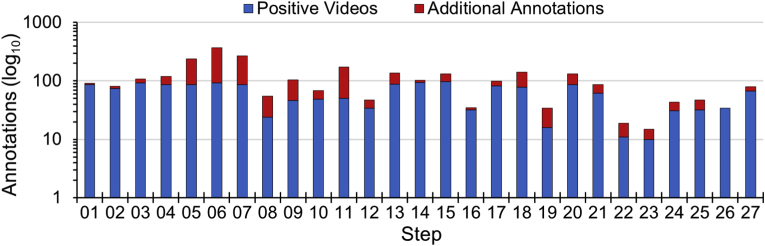
A bar chart of the annotations across all 97-videos per surgical step. “Positive Videos” indicates the number of videos where a step is annotated at least once, and adding this to “Additional Annotations” indicates the total number of annotations. Note the annotations scale is logarithmic base 10.

The nomenclature for a video containing an annotated step is called a “positive video” and otherwise a “negative video”. Similarly, a frame considered to contain a step is called a “positive frame” and otherwise a “negative frame”. The distribution of annotations across all 97-videos per step is displayed in Fig. 1, where a wide range of annotation numbers are seen. The total number of annotations correlates with the number of positive videos, with some steps having several more annotations. Focusing on positive videos, core steps (e.g. S15) are present in the majority of the videos, whereas optional steps (e.g. S23) and instrument steps (e.g. S19) are in far fewer videos.

20-videos were randomly chosen as the testing dataset, and fixed for all stages in the classification architecture. For the remaining 77-videos, in order to account for the imbalance in the step distribution of positive videos, the training and validation videos changed depending on the step being classified and stage of the classification architecture, as described in the next section.

### 2.2 Surgical step classification

**Fig. 2:**
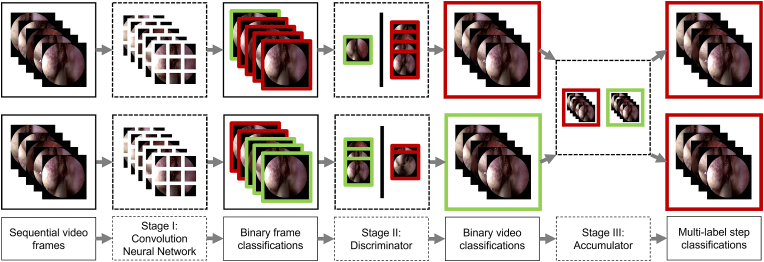
A flow diagram representation of the architecture used to automatically generate the operation notes. In stage I: for each step, a convolution neural network is used as a binary image classifier on each frame of a video. In stage II: for each step, a discriminator is used as a binary video classifier. In stage III: for each video, an accumulator is used as a multi-label step classifier. In this diagram, a green outline represents a positive classification and a red outline represents a negative classification. Note in this representation, only 2-steps (S02 and S03) are classified, in the full version, all 27-steps are classified. (For interpretation of the references to colour in this figure legend, the reader is referred to the Web version of this article.)

The aim is to automatically detect which of the 27-steps are present in a given video. To achieve this, a three-stage architecture was created, as displayed in Fig. 2. The three-stages are trained in sequence, with Stage I completing hyperparameter tuning on the validation dataset before Stage II is trained. All code is written in Python 3.8^2^ [15], using PyTorch 1.8.1^3^ [16], will be publicly available,^4^ and run on a NVIDIA Tesla V100 Tensor Core 32 GB GPU using CUDA 11.2^5^ [17].

#### 2.2.1 Stage I: Binary frame classification (CNN)

**Fig. A1:**
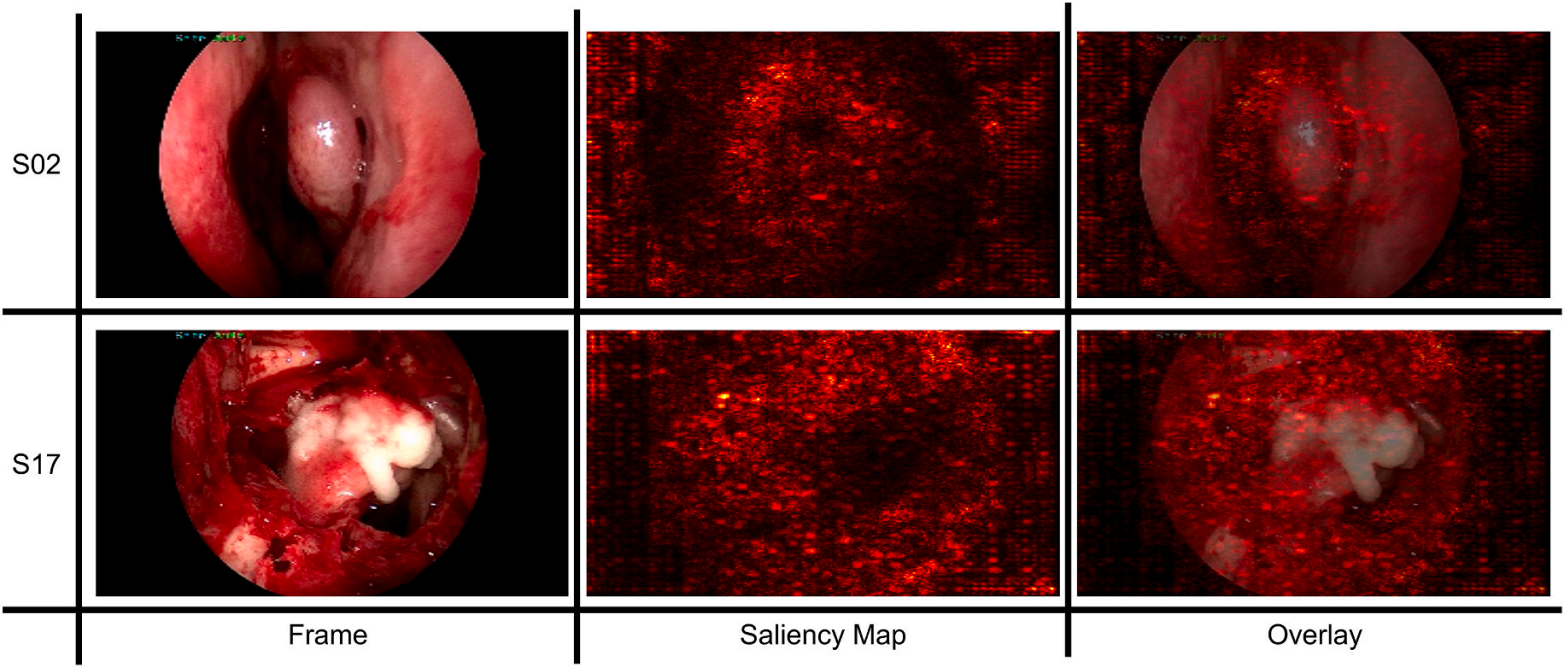
ResNet50 saliency maps for two example steps within one video. The steps displayed are S02 (identification of the sphenoid ostium, Phase 1) and S17 (haemostasis, Phase 3). Saliency maps highlight the pixels, and by proxy the features, deemed to be important by ResNet50 for a positive classification.

The first stage is to create a binary frame classifier for each step. Frame-level 7-steps multi-class classification has been previously investigated on a 50-videos subset of this 97-videos dataset, where the optimal CNN was found to be ResNet50 [11,18]. For a given step, ResNet50 is able to distinguish between a positive and negative frame by identifying the key features within the image. For example, for S02 (identification of the sphenoid ostium, Phase 1) ResNet50 will identify biological landmarks that differ from the other steps. Alternatively, for S17 (haemostasis, Phase 3), a repair technique will contrast the usual biological landscape, and this will be identified and differ from the other steps. An example of a saliency maps for these two example steps (S02, S17), where features are highlighted via a heatmap, are displayed in Appendix Figure A1.

Hence, the chosen CNN is ResNet50 pre-trained on ImageNet, with the final layer replaced with a linear classifier and the softmax activation function. The loss function is cross-entropy, and the optimiser is stochastic gradient descent with a learning rate of 0.001 and momentum 0.9, run with a batch size of 8 for 8 epochs. Training images were randomly resized; randomly cropped; and randomly horizontally flipped (validation images remain unchanged), before both training and validation images were colour normalised and resized to 224 × 224 pixels to match the ImageNet dataset. The epoch with the highest weighted-*F*_1_ score on the validation dataset was kept, as weighted-*F*_1_ score safeguards against both small precision and small recall. A summary of the parameter/hyperparameter values used for training can be found in Appendix Table A1.

As not all of the 77-videos in the non-test dataset contain every step, for a given step, only positive videos were used for training and validation. An approximate 4-training to 1-validation random split was used, and ResNet50 was trained and validated on both positive and negative frames of the training and validation dataset respectively. For each annotated step, the current and following frames until the next non-instrument step were defined as positive frames. As negative frames outnumber positive frames, to prevent class imbalance, the number of negative frames were reduced to the match the number of positive frames. This was done by randomly choosing negative frames from a positive video until the number of positive frames and negative frames in the same video match. Frames from a negative video were not used for training or validation.

For the discriminators used in stage II, all frames need to be classified. For this evaluation, frames for all 97-videos were colour normalised and resized to 224 × 224 pixels to match the ImageNet dataset. Then, the frames were classified using the best performing CNN for that respective step, outputting both the binary classifications and the frame classification probabilities (before the 0.5 threshold for binary classification) as temporal-ordered sequences.

#### 2.2.2 Stage II: Binary video classification (discriminator)

The second stage is to create a binary video classifier for each step. To achieve this, six custom discriminators were created and trained for each step. These discriminators were inspired by temporal smoothing functions, which have been shown to improve the performance of 7-step multi-class classifications when applied to CNN classifications on the 50-videos subset of this 97-videos dataset [11]. All discriminators are based on calculating a single “discrimination number” (*δ*) from a videos’ temporal-ordered sequential frame classifications and if *δ* is greater than or equal to a “discrimination threshold” (*τ*), that video is predicted to be positive. A list of the discriminators and the calculation for their respective discrimination number is given in Table 2.

**Table 2:** Definitions of the six discriminators created and trained for stage II. A discriminator predicts a positive video if the discrimination number is greater than or equal to the discrimination threshold (i.e. *δ* ≥ *τ*). The threshold stride and maximum values are also given. Note 16200 is the maximum discrimination threshold for integer-discriminators as the longest video in the training dataset has 16103 frames.

Discriminator name	Discrimination number (*δ*)	Discriminator threshold (*τ*)	
		Stride	Maximum
Binary integer	*∑*positive frames	100	16200
Binary fraction	*∑*positive frames/*∑*frames	0.01	1
Probability integer	*∑*frame probabilities	100	16200
Probability fraction	*∑*frame probabilities/*∑*frames	0.01	1
Chain integer	longest period of positive frames	1	16200
Chain fraction	longest period of positive frames/*∑*frames	0.001	1

For each discriminator, *τ* is a hyperparameter to be determined. This was done via linear grid search starting from a minimum of *τ* = 0 for all discriminators, with varying maximums and strides for each discriminator, as given in Table 2. Weighted-*F*_1_ score is the chosen evaluation metric as it accounts for the imbalanced dataset, and the value of *τ* that maximises the weighted-*F*_1_ score was set as the optimal *τ* value. A validation dataset is not required as *τ* is found in a singular pass, and so the training dataset was on all 77-videos in the non-testing dataset. For the accumulator used in stage III, for a given video, classifications are required for all steps. For this evaluation, for each step, a video was classified based on the respective step's optimal value of *τ*, and then all step classifications for that video were outputted as a step-ordered sequence.

#### 2.2.3 Stage III: Multi-label steps classification (accumulator)

The third stage is to create a multi-label steps classifier for each video. By taking the step-ordered classification sequence from stage II, an “accumulator” was created to ensure the relationships between steps do not contradict one another. These rules were created by clinical consensus and are given in the “Reliant” column in Table 1 or more clearly stated in Appendix Table A3. For example, S11 (“confirmed using neuronavigation”) cannot have a positive classification unless either S09 or S10 (“The sella, carotid prominence, optic prominence, and optic-carotid recesses were then identified on both sides”) also has a positive classification. If the discriminator positively predicts S11 but does not positively predict S09 or S10 then S11 is changed to a negative prediction. The finalised step classifications were then inputted into the operation notes template.

**Table 3:** 7-steps mean-averaged weighted-*F*_1_ scores for the six discriminators, before and after the use of the accumulator. Scores are given to two significant figures with standard deviation. Ensemble discriminator results can be found in Appendix Table A4.

Discriminator name	Discriminator		Accumulator	
	Training	Testing	Training	Testing
Binary integer	0.72 ± 0.15	0.73 ± 0.24	0.71 ± 0.15	0.73 ± 0.23
Binary fraction	0.77 ± 0.13	0.77 ± 0.21	0.76 ± 0.13	0.77 ± 0.18
Probability integer	0.72 ± 0.15	0.73 ± 0.24	0.71 ± 0.15	0.73 ± 0.23
Probability fraction	0.76 ± 0.15	0.79 ± 0.20	0.76 ± 0.12	0.80 ± 0.18
Chain integer	0.60 ± 0.21	0.61 ± 0.27	0.58 ± 0.21	0.60 ± 0.26
Chain fraction	0.69 ± 0.19	0.72 ± 0.26	0.68 ± 0.18	0.72 ± 0.25

### 2.3 Operation notes template

**Fig. 3:**
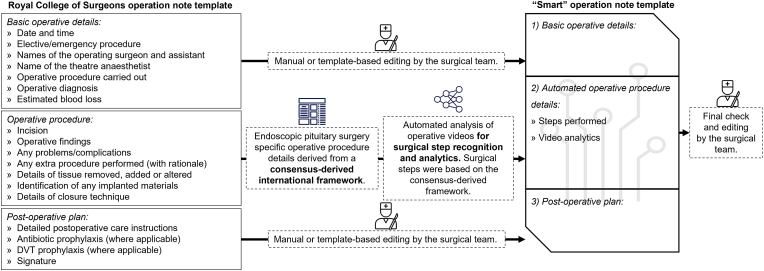
Flow diagram of how the automatically generated “smart” operation notes template is intended to be created. The template is derived from three sources: the Royal College of Surgeons [19], an international Delphi consensus study [14], and manual editing from surgical teams. Note final checks are always to be completed by the surgical team.

The intended automatically generated “smart” operation notes template, as displayed in Fig. 3, was derived from three sources. Firstly, the Royal College of Surgeons in England Good Surgical Practice guidelines^6^ were used to define the minimum information set required for each operative note: basic information, surgical procedure, and post-operative plan [19]. Secondly, an existing international expert consensus [14] was used to define the components of the surgical procedure for endoscopic pituitary surgery: phases; steps; instruments; and errors. In this section, the text is automated from the steps classifications output from the described three-stage architecture and the video analytics (3-phases duration chart) from the preexisting multi-class classifications from the architecture described in Ref. [11]. Thirdly, any remaining sections of the operative note would require template-based or manual editing by surgical teams.

## 3 Results and analysis

### 3.1 Stage I: CNN

**Fig. 4:**
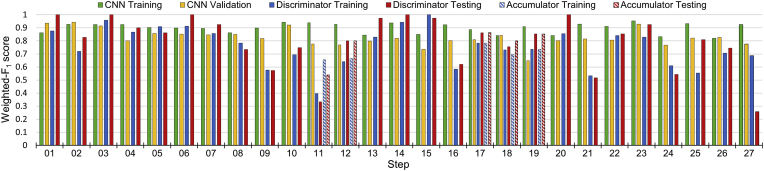
A bar chart of the weighted-*F*_1_ scores for all three-stages across the 27-steps, as displayed in the legend. Stage I: CNN (ResNet50) binary frame classifications; Stage II: Discriminator (probability fraction) binary video classifications; Stage III: Accumulator multi-label classifications applied to discriminator classifications for reliant steps (see Table 1).

Fig. 4 displays the weighted-*F*_1_ for all three-stages across the 27-steps. Focusing on CNN (stage I), for each step the first bar (green) displays the training dataset weighted-*F*_1_ score and the second bar (yellow) the validation dataset weighted-*F*_1_ score. The mean-average weighted-*F*_1_ across all 27-steps is 0.90±0.04 and 0.83±0.06 respectively. It is found that for a given step, ResNet50 is able to distinguish between the positive and negative frames with high performance, with a reasonable training to validation dataset translation. S19 (bipolar cutlery, instrument), has a particularly low weighted-*F*_1_ score of 0.65 on the validation dataset, although a high score of 0.91 on the training dataset. This poor translation is likely due to the small number of positive videos (see Fig. 1), leading to high variability in images.

### 3.2 Stage II: Discriminator

The “Discriminator” column in Table 3 displays the weighted-*F*_1_ scores for the six discriminators, mean-averaged across the 27-steps for both training and testing datasets. The performances are high, with the best performing discriminator on the training dataset (binary fraction) scoring 0.77, and the best on the testing dataset (probability fraction) scoring 0.79. It is found that fraction-based discriminators outperform integer-based discriminators, likely due to the variability in the video lengths. It is also found that chain-based discriminators are worse performing than both probability-based and binary-based discriminators (which perform similarly). This is likely due to “step flickering” phenomena found in both [11,13], where occasionally a frame is seemingly randomly predicted a negative class when it is truly positive, due to occlusions of the endoscope/laparoscope. Hence a “chain” of positive frames breaking, and not leading to a consistent discrimination number.

The weighted-*F*_1_ scores translate well from the training dataset to the testing dataset, with almost identical performance (although slightly higher standard deviations in the testing dataset), implying generalisability of the discriminators. The weighted-*F*_1_ scores for the 27-steps for the best performing discriminator, probability fraction, can be found in Fig. 4. The third bar (blue) displays the training dataset weighted-*F*_1_ and fourth bar (red) displays the testing dataset weighted-*F*_1_. Focusing on the step-specific performance, for the majority of the steps the training weighted-*F*_1_ score is high (>0.75), yet there is some score variability: e.g. S15 (tumour excision, core) has a score of 1.0 whereas S11 (stealth pointer, instrument) has a score of 0.40. Additionally, the weighted-*F*_1_ score is stable against small changes in the optimal discrimination threshold (*τ*±0.05). The optimal values for *τ* can be found in Appendix Table A2. The results for ensemble discriminators, created by combining the classifications of probability fraction with the remaining 5-discriminators with either an intersection (∩, and) or union (∪, or) operator, can be found in Appendix Table A4. The weighted-*F*_1_ score for these ensemble discriminators show no improvement over the baseline probability fraction discriminator weighted-*F*_1_ score for both training and testing datasets.

### 3.3 Stage III: Accumulator

The “Accumulator” column in Table 3 displays the weighted-*F*_1_ scores for the six discriminators after the accumulation stage, mean-averaged across the 27-steps for both training and testing datasets. On both datasets, the weighted-*F*_1_ score is changed by approximately 0.01 in all cases, with a similarly small change in the standard deviation, showing that the accumulator has minimal impact on the classifications. This is expected as there are relatively few rules where the step classifications outputted by the discriminator will change.

This observation is repeated in Fig. 4, where the weighted-*F*_1_ scores across the 27-steps are displayed after the accumulator has been applied to the probability fraction discriminator binary video classifications when reliant (see Table 1). The fifth bar (dashed blue) displays the training dataset weighted-*F*_1_ score and sixth bar (dashed red) displays the testing dataset weighted-*F*_1_ score. The only major changes are a decreased performance in S03 (anterior sphenoidotomy, core) and an increased score in S11 (stealth pointer, instrument). The direction of changed performance is in the direction of the performance of the reliant step at the discrimination stage. Specifically (see Table 1), S03 is reliant on S02 which has a worse performance and S11 is reliant on S09 or S10 which both have better performances.

## 4 Discussion

### 4.1 Principal findings

In this paper, a novel three-stage architecture was created and trained on a 97-videos dataset of endoscopic pituitary surgery, in order to create a multi-label steps classifier which determines which surgical steps are present within a given video. The three-stages were: (I) binary frame classification; (II) binary video classification; (III) multi-label steps classification. For stage I, the CNN ResNet50 was shown to be an effective binary frame classifier. For stage II, several novel discriminators were created, and it was shown binary fraction was the most effective binary video classifier. This binary fraction discriminator classifies a video as positive based on whether the fraction of positive frames (the discrimination number) is greater than or equal to a certain number (the discrimination threshold). For stage III, a custom accumulator ensured clinical coherence between the surgical steps using predetermined rules, while having minimal impact on step classification performance. Operation notes were further enriched with a 3-phases duration chart using preexisting methods. An example of an automatically generated smart operation note for a specific video is given as supplementary material.

### 4.2 Comparison to literature

Step recognition from videos has well-established methods: CNNs are used for spatial recognition and recurrent neural networks or temporal-CNNs for temporal recognition [10]. Using this as a basis, for stage I of the three-stage architecture, ResNet50 was chosen as the binary frame classifier. This is because it was shown to be the optimal CNN in Ref. [11], where 0.67 weighted-*F*_1_ score was achieved in 7-steps multi-class recognition on a 50-videos subset of the 97-videos used in this study. The 0.83 weighted-*F*_1_ score achieved by ResNet50 in this case is therefore comparable, and an improved performance is expected considering the simpler binary classification task. Temporal recognition via neural networks was not introduced in this study given the duration for each step is short, leading to a small positive dataset, and the need for multiple consecutive frames would reduce this dataset further. Statistical methods for utilising temporal sequencing, such as temporal smoothing as used in Ref. [11], was introduced in stage II of the architecture through the use of discriminators.

[13] provided a proof of concept for automatic generation of operation notes through the use of 8-phases classification on laparoscopic cholecystectomy. In that study, a spatial-temporal CNN was trained on 52-videos and tested on 15-videos where a 0.80 accuracy was achieved. This is comparable to the 0.80 weighted-*F*_1_ score achieved in this paper's study, but with multi-labelled 27-steps compared to far fewer single-labelled 8-phases.

### 4.3 Strengths and limitations

One strength of this study is the large 97-videos dataset, although it is from a single centre which means the architecture's true generalisability is not known. Collecting more videos across many different centres will improve this.

Another strength is the number of novel discriminators used, and their effectiveness as a binary video classifier. However, they perform less well on steps where the number of positive and negative frames are similar, due to the threshold technique implemented. More granular discriminators will help fix this particular issue. Moreover, pretraining ResNet50 on multi-class 3-phases and 7-steps frame classifications [11] or instrument classifications may improve the binary frame step classifications, which may help improve performance at the discrimination stage. This may also supersede the need for binary classifications and move straight to 27-steps multi-label step classifications. However, given the current relatively small dataset size; the small number of videos for certain steps (e.g. S23 is found in only 15 videos); and short duration of some steps (e.g. S04 is <60s on mean-average), more data and more sophisticated classifiers are required. After this, more sophisticated discriminators can be used for multi-step video classification.

A third strength is the use of an accumulator to ensure clinical coherence between steps, which has been shown to be effective. A limiting factor for this is the static nature of the rules. The use of step transition probabilities to modify predictions through the use of statistical methods, such as hidden Markov models, to create dynamic rules may improve the accumulator's performance in stage III multi-label steps classification.

Finally, the operation note template is derived from domestic surgical standards and international consensus studies, which is a strength. To improve this further, images of critical steps (S09, S10, S15, S16, S21) would be added as a video analytic, in addition to the 3-phases duration chart. Furthermore, the notion of step flickering could be used to flag a surgery that has deviated from the norm, as in Ref. [13].

### 4.4 Clinical translation

Although the run-time for training is approximately 12 hours, the evaluation of a singular video is less than 2 minutes, thus the operation note statements can be generated immediately after a surgery. Next comes the clinical validation of the generated notes, and if deemed successful, they should be implemented into clinical workflow. Here the true benefits of automation will be seen; increasing operation notes accuracy and granularity while reducing the administrative burden on clinicians [4,20]. The automatically generated notes will require final sign-off from the surgical team. Therefore, if clinicians are uncomfortable with a purely automatically generated operation note, the predicted steps can be used as a prompt for manually written operation notes, and ensure steps are not missed.

In order to translate this methodology to other surgeries, the following steps are suggested:

1. The surgery should be broken down into well-defined surgical steps. It is recommended this is achieved via an international Delphi consensus study [14].
2. Each step should then have an associated operative statement created, with reliant steps accounted for (e.g. Table 1). It is recommended guidance from the appropriate governing medical body is adhered too [19].
3. Several surgeries should be recorded. It is recommended to record over 50 multi-centered videos for improved generalisability [11].
4. Each surgical video should then be annotated with the timestamps of the start and end of each surgical step. This will me a manual process, and require the use of clinicians with experience in the surgery [12].
5. The three-stage architecture created in this paper should then be trained on annotated videos. It is recommended hold-out testing is used to ensure a certain quantitative value is achieved before putting this into clinical practice.
6. Finally, after a new surgery is completed, the video of the surgery can be fed into the trained model, and the model output will automatically generate the operation note.

### 4.5 Conclusion

In this paper, it was shown that automatic generation of operation notes from endoscopic pituitary videos using workflow recognition is possible to achieve with a high performance and efficiency. This is the first such automatic generation of operation notes in endoscopic pituitary surgery, and on top of this, the operation notes are enriched with video analytics. Hence, this work paves the way for future endeavours in the clinical translation of step recognition to the automatic generation of operation notes, where it can be used as an assistive tool by clinicians, reducing their administrative burden.

## Funding

This research was funded in whole, or in part, by the 10.13039/100004440Wellcome/EPSRC Centre for Interventional and Surgical Sciences (WEISS) [203145/Z/16/Z]; the Engineering and Physical Sciences Research Council (EPSRC) [EP/P027938/1, EP/R004080/1, EP/P012841/1]; and the Royal Academy of Engineering Chair in Emerging Technologies Scheme. AD is supported by 10.13039/501100000266EPSRC [EP/S021612/1]. HJM is supported by WEISS [NS/A000050/1] and by the National Institute for Health and Care Research (NIHR) Biomedical Research Centre at 10.13039/501100000765University College London. DZK is supported by the NIHR Academic Clinical Fellowship and the Cancer Research UK (CRUK) Predoctoral Fellowship. JH is supported by an NIHR Academic Clinical Fellowship. For the purpose of open access, the author has applied a CC BY public copyright licence to any author-accepted manuscript version arising from this submission.

## Ethical approval

This article does not contain any studies with human participants or animals performed by any of the authors.

## Consent

The study was registered with the National Hospital for Neurology and Neurosurgery local audit committee and data sharing was approved by the information governance lead. All patients provided written informed consent for their images to be collected for research.

## Data, code and/or material availability

The data is not publicly available. The code is publicly available at https://github.com/dreets/pitnet-opnotes-public. A completed smart operation note is given as supplementary material.

## Declaration of competing interest

The authors declare that they have no known competing financial interests or personal relationships that could have appeared to influence the work reported in this paper.

## Figures and Tables

**Table A1:** Convolution neural network binary classification parameter choice. No hyperparameter tuning was done - values are taken from the most optimal convolution neural network found in Ref. [11].

Parameter	Value
Dataset size	97-videos
Mean length	77.9 min
Validation	4 to 1 training to validation split
Testing	20-videos hold-out testing
Class imbalance	Equal number of positive and negative frames for training
Encoder	ResNet50 (pre-trained on ImageNet)
Classification activation function	Softmax
Loss function	Cross-entropy
Optimiser	Stochastic gradient descent
Learning rate	0.001
Momenumtum	0.9
Batch size	8
Number of epochs	8
Image size	224 × 224 pixels
Training image augmentation	Random cropping; random horizontal flipping; colour normalisation
Primary evaluation metric	Weighted-*F*_1_ score

**Table A2:** Threshold number (*τ*) values for the 27-steps for all discriminators. Definitions of each discriminator can be found in Table 2 of the main paper. The value of *τ* was tuned by trying values between 0 and the displayed maximum, increasing *τ* by the displayed stride at each iteration. The value that maximises the weighted-*F*_1_ score across all 77-non-test-videos for a given step is displayed here.

Discriminator	Binary integer	Binary fraction	Probability integer	Probability fraction	Chain integer	Chain fraction
Stride	00100	0.01	00100	0.01	00001	0.001
Maximum	16200	1.00	16200	1.00	16200	1.000
S01	1500	0.05	1500	0.05	158	0.001
S02	0700	0.05	0700	0.04	156	0.001
S03	1500	0.05	1500	0.05	151	0.001
S04	1500	0.05	1500	0.05	159	0.001
S05	1500	0.05	1400	0.05	159	0.001
S06	1500	0.05	1500	0.05	158	0.001
S07	1500	0.10	1400	0.10	160	0.001
S08	0000	0.00	0000	0.00	046	0.000
S09	0300	0.09	0300	0.09	080	0.000
S10	0300	0.09	0300	0.09	159	0.001
S11	0300	0.09	0300	0.09	095	0.001
S12	0100	0.09	0100	0.09	094	0.000
S13	1500	0.10	1400	0.10	157	0.001
S14	1500	0.10	1500	0.10	161	0.001
S15	1100	0.10	1100	0.10	159	0.001
S16	0400	0.08	0400	0.08	087	0.000
S17	1500	0.10	1400	0.10	139	0.001
S18	1300	0.09	1200	0.09	151	0.001
S19	0100	0.08	0000	0.07	022	0.000
S20	1500	0.10	1400	0.10	149	0.001
S21	0700	0.09	0700	0.10	090	0.001
S22	0100	0.08	0000	0.07	220	0.000
S23	0000	0.00	0000	0.00	017	0.000
S24	0200	0.08	0200	0.09	012	0.000
S25	0100	0.09	0100	0.08	026	0.000
S26	0200	0.07	0200	0.07	078	0.000
S27	1500	0.10	1500	0.09	160	0.001

**Table A3:** The rules for the accumulator. A listed “predicted step” cannot be positively predicted without the “reliant step” also being positively predicted. If this is the case, the “predicted step” is changed to a negative prediction. Step statements can be found in Table 1 of the main paper and step definitions can be found in Ref. [14].

**Predicted Step**	S03	S11	S12	S17	S18	S19
**Reliant Step**	S02	S09 or S10	S09 or S10	S18 or S19 or S20	S17	S17

**Table A4:** 27-steps mean-averaged weighted-*F*_1_ scores for ensemble discriminators. The ensemble discriminators are created by combining the best performing baseline discriminator classifications, probability fraction, with the remaining 5-discriminators’ classifications, using either an intersection (∩, and) or union (∪, or) operator.

Ensemble Discriminator	Weighted-*F*_1_ score	
	Training	Testing
Probability fraction	0.76 ± 0.15	0.79 ± 0.20
Probability fraction ∩ Binary integer	0.72 ± 0.16	0.72 ± 0.17
Probability fraction ∪ Binary integer	0.73 ± 0.14	0.73 ± 0.15
Probability fraction ∩ Binary fraction	0.74 ± 0.13	0.74 ± 0.15
Probability fraction ∪ Binary fraction	0.74 ± 0.14	0.74 ± 0.16
Probability fraction ∩ Probability integer	0.74 ± 0.14	0.74 ± 0.16
Probability fraction ∪ Probability integer	0.65 ± 0.25	0.66 ± 0.25
Probability fraction ∩ Chain integer	0.62 ± 0.15	0.62 ± 0.15
Probability fraction ∪ Chain integer	0.63 ± 0.15	0.61 ± 0.15
Probability fraction ∩ Chain fraction	0.72 ± 0.17	0.72 ± 0.18
Probability fraction ∪ Chain fraction	0.70 ± 0.18	0.71 ± 0.19

## Footnotes

2 https://www.python.org/.

3 https://pytorch.org/.

4 https://github.com/dreets/pitnet-opnotes-public.

5 https://developer.nvidia.com/cuda-toolkit.

6 https://www.rcseng.ac.uk/standards-and-research/gsp/.
