# Supplementary material for: Automatic generation of operation notes in endoscopic pituitary surgery videos using workflow recognition
Source: Intell Based Med. 2023;8:100107. doi: 10.1016/j.ibmed.2023.100107 (PMC10958393; doi:10.1016/j.ibmed.2023.100107)
Supplement: Multimedia component 1 [file mmc1.pdf]

Basic operative details

<u>Smart Operation Note</u>

Name: -
DOB: -
Hospital number: -
NHS number: -

Op date/time: -
Surgeon(s) and Role:

- -
- -
- -

Named Consultant Anaesthetist: -
Anaesthetist: -
Anaesthetist Type: General

Procedure(s):
Endoscopic Transsphenoidal Excision of Pituitary Adenoma (Cushing’s) +/- Fat Graft +/- Lumbar Drain

Anaesthesia
The patient was transferred to the operating room and given preoperative prophylactic IV antibiotics. Moffett’s solution (10ml) was used to prepare the nasal mucosa.

Positioning
The patient was placed supine with their head in a Mayfield clamp, and their head slightly flexed and rotated in a ‘conversational’ pose. All pressure points were well padded. The patients nose and the left lower abdomen was then prepared and draped in the usual fashion. Neuronavigation was set up and its accuracy confirmed.

Automated operative procedure details

Incision and exposure
A short 0 degree endoscope was used to visualise nasal cavity and the choana identified. Through a right mononostril approach, the middle and superior turbinates were laterally displaced using a freer elevator. The sphenoid ostium was identified and opened using Kerrison's rongeurs. The mucosa inferior aspect of the ostium was carefully stripped, protecting the sphenopalatine artery. The septum was then displaced and a partial posterior septectomy performed until the opposite ostium was seen. The sphenoid sinus was opened, with removal of sphenoid septations and mucosa to expose the face of the sella. The endoscope was then fixed using a holder. The sella was carefully opened using Kerrison rongeurs. A cruciate durotomy was performed using a retractable scalpel.

Findings
The tumour was seen immediately on entering the sella and removed in a piecemeal fashion using currettes and pituitary rongeurs. Aliquots of saline were injected in into the lumbar drain to promote descent of the suprasellar component. The cleared pituitary fossa was visualised, and the diaphragm had descended. An arachnoid tear was noted.

Closure
Haemostasis was achieved with a spongostan placement.

Video analytics

- Phases duration chart: Phase 1 (Nasal); Phase 2 (Sellar); Phase 3 (Closure).

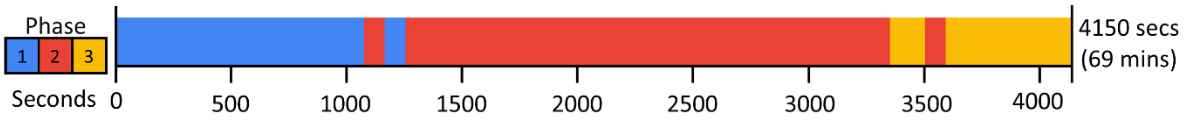

Post-operative plan

Post-op instructions

- Daily fluid balance and U&E; watch Na (sodium) levels, in particular. Please discuss with pituitary-, endocrine-, or anaesthetic registrar before giving DDAVP.
- Daily 9am cortisol levels, and if serum cortisol is less than 50nM please start hydrocortisone 20mg tds orally.
- Can mobilise as tolerates, no staples to remove post-op.
- Please ensure TED stockings and Flowtron boots, and can start prophylactic LMWH from the evening of post-op day 1.
